# Molecular Basis of Taste and Micronutrient Content in Kumamoto Oysters (*Crassostrea Sikamea*) and Portuguese Oysters (*Crassostrea Angulata*) From Xiangshan Bay

**DOI:** 10.3389/fphys.2021.713736

**Published:** 2021-07-27

**Authors:** Sheng Liu, Hongqiang Xu, Shoushuo Jian, Qinggang Xue, Zhihua Lin

**Affiliations:** ^1^Institute of Mariculture Breeding and Seed Industry, Zhejiang Wanli University, Ningbo, China; ^2^Zhejiang Key Laboratory of Aquatic Germplasm Resource, Zhejiang Wanli University, Ningbo, China

**Keywords:** nutrients, taste, Kumamoto oyster, Portuguese oyster, sympatric distribution

## Abstract

Oysters are the most extensively cultivated bivalves globally. Kumamoto oysters, which are sympatric with Portuguese oysters in Xiangshan bay, China, are regarded as particularly tasty. However, the molecular basis of their characteristic taste has not been identified yet. In the present study, the taste and micronutrient content of the two oyster species were compared. Portuguese oysters were larger and had a greater proportion of proteins (48.2 ± 1.6%), but Kumamoto oysters contained significantly more glycogen (21.8 ± 2.1%; *p* < 0.05). Moisture and lipid content did not differ significantly between the two species (*p* > 0.05). Kumamoto oysters contained more Ca, Cu, and Zn (*p* < 0.05); whereas Mg and Fe levels were comparable (*p* > 0.05). Similarly, there was no significant difference between the two species with respect to total amount of free amino acids, umami and bitterness amino acids, succinic acid (SA), and most flavoring nucleotides (*p* > 0.05). In contrast, sweetness amino acids were significantly more abundant in Portuguese oysters. Volatile organic compounds profiles of the two species revealed a higher proportion of most aldehydes including (2*E*,4*E*)-hepta-2,4-dienal in Kumamoto oysters. Overall, Kumamoto oysters contain abundant glycogen, Ca, Zn, and Cu, as well as volatile organic compounds, especially aldehydes, which may contribute to their special taste. However, free amino acid and flavor nucleotides may not the source of special taste of Kumamoto oyster. These results provide the molecular basis for understanding the characteristic taste of Kumamoto oysters and for utilizing local oyster germplasm resources.

## Introduction

Oysters are found throughout the world, where they constitute an important marine resource and the most cultivated bivalves. Numerous oyster farms exist in China and each region are characterized by a different dominant species (Wang et al., 2008, 2010). Recently, we described how the Kumamoto oyster (*Crassostrea sikamea*), a cryptic indigenous species (Liu et al., 2021), comprised more than 70% of the oyster samples cultivated along the Zhejiang coast. Accordingly, at least 50,000 tons of the local annual oyster production is likely composed of *C. sikamea*, making this the world’s largest Kumamoto oyster farming area.

Kumamoto oysters grow naturally in the warm seas of East Asia, from southern Japan to Vietnam (Banks et al., 1994; Hamaguchi et al., 2013; Wang et al., 2013; In et al., 2017). Even though in the 1940s they were mistaken as Pacific oysters from the Ariake Sea in Japan and introduced to the west coast of the United States, their global production remains limited (Sekino, 2009). Kumamoto oysters are relatively small compared to other *Crassostrea* oysters; individuals cultured in Zhejiang Province weight no more than 30 g. They also appear to be sympatric with Portuguese oysters (*Crassostrea angulata*) along the southern coast of China (Wang and Guo, 2008; Xia et al., 2009).

Kumamoto oysters tend to be deeper cupped and are regarded as tastier compared to other cultured species, because their meat retains a firm texture even during the summer season (Sekino, 2009) and according to local oyster farmers, *C. sikamea* seems to have better taste and special flesh color. Oysters are rich in protein, glycogen, free amino acids, polyunsaturated fatty acids, volatile organic compounds, trace elements, and minerals; moreover, they are highly edible and are purported to have medicinal properties (Dridi et al., 2007; Pogoda et al., 2013; Yuasa et al., 2018). The nutrient composition of different oyster species has been extensively studied in many areas (Oliveira et al., 2006; Pogoda et al., 2013; van Houcke et al., 2016; Qin et al., 2018; Yuasa et al., 2018; Zhu et al., 2018; Lin et al., 2019). Biochemical characteristics, volatile organic compounds, taste, and micronutrients vary among species (van Houcke et al., 2016; Yuasa et al., 2018; Lin et al., 2019), strains or brands of the same species (Zhu et al., 2018; Murata et al., 2020), tissues (Liu et al., 2013, 2020), ploidy levels (Qin et al., 2018), and even culture conditions (Pennarun et al., 2003; van Houcke et al., 2017; Bi et al., 2021). While Kumamoto oysters seem to have a unique taste (Lin et al., 2019), the molecular basis underlying this characteristic remains unknown. Understanding which micro- or macronutrients are related to taste determination may direct operators toward more targeted resource utilization and food processing methodologies.

In this study, general nutrients and mineral components, of which glycogen may contribute to sweet taste, Ca, Zn, Cu contribute to its mineral nutritional value, free amino acids, succinic acid (SA), nucleotides, and related compounds, which are thought to be responsible for its umami as well as volatile organic compounds, which represent the characteristic odors of the two oyster species cultivated in Xiangshan bay (i.e., Kumamoto and Portuguese oysters) were measured and compared in order to find substances related to Kumamoto oyster unique taste and odor. Differences in the chemical properties of the two oyster species are systematically discussed. These results will improve our understanding of the molecular basis underlying the special taste of Kumamoto oysters and the utilization of local oyster germplasm resources.

## Materials and Methods

### Sample Collection and Growth Performance Measurement

Cultured oyster samples were collected from Xiangshan bay (29.47 N, 121.42 E), Ningbo City, where it was the dominant oyster species in the local bay. Oysters were sampled at the 20-month-old in February when the oysters have not started the gametogenesis and reach a commercial size. One hundred individuals each of *C. angulata* and *C. sikamea* were collected. According to a preliminary classification, the flesh of *C. sikamea* was yellow-green; whereas the flesh of *C. angulata* was white. The growth performance of the two species was evaluated by measuring shell height, length, and width using a vernier caliper (sensitivity = 0.01 mm). An electronic balance (sensitivity = 0.01 g) was used to measure total weight (weight before shucking the oyster), soft weight (weight of soft body), and dry soft weight. Dry soft weight was measured after freeze-drying for 48 h; whereas the shells were oven-dried at 80°C for 48 h. The condition index was calculated as the percentage of soft weight relative to dry soft weight, the dressing percentage was calculated as the ratio of dry flesh weight relative to dry shell weight (Liu et al., 2020). The oysters were shucked, and the adductor muscle was selected for DNA extraction and subsequent species identification by specific molecular methods (Liu et al., 2021) and the results are summarized in Supplementary Figure 1.

### Proximate Composition

A total of 100 individuals were used for whole-body measurement and whole-flesh nutrient analysis for each of the species. Prior to nutrient analysis, the flesh was freeze-dried for 48 h and then ground into powder using a tissue grinder (Jingxin, Shanghai, China) in the presence of liquid nitrogen. Moisture content was determined individually from the difference between soft weight and dry soft weight divided by soft weight. For latter nutrients measurements, each sample consisted of the powdered flesh of 25 oysters, and each data point represented the mean of four replicates.

Glycogen content was measured using a commercial kit based on the anthrone colorimetry method with glucose as standard (Jiancheng, Nanjing, China) and the glycogen content was calculated accordingly the manufactures instruments. Protein content was analyzed using a Kjeldahl analyzer (KD310; OPSIS AB, Furulund, Sweden) and protein content was calculated by multiplying the nitrogen content by 6.25 (Qin et al., 2018). Crude lipid was determined using the Soxhlet extraction method (2055 Soxtec Avanti; Foss, Hilleroed, Denmark) and petroleum ether as the extractor. Glycogen/protein/lipid content were measured as mg/g of dry weight and shown as percentage of dry weight finally. Ca, Mg, Cu, Zn, Fe, and Mn were measured using an inductively coupled plasma-optical emission spectrometer (ICP-OES, 5110; Agilent Technologies, CA, United States) at the State Key Laboratory of Food Science and Technology at Jiangnan University, Wuxi, China, and were expressed as mg/kg relative to dry weight.

### Taste-Related Substance Determination

Free amino acids, nucleotides and their related compounds, and SA were measured using high-performance liquid chromatography (HPLC) with prepared dried powder and performed at the State Key Laboratory of Food Science and Technology at Jiangnan University, Wuxi, China.

The free amino acids were extracted using a method previously reported by Murata et al. (2020). Around 1.00 g of oyster powder was homogenized in 10 ml of 5% (v/v) trichloroacetic acid and precipitated for 2 h. The homogenate (1 ml) was centrifuged at 10,000 rpm for 15 min and 0.4 ml of the supernatant was decanted to a new tube. Its pH was adjusted to 2, the volume was set to a constant value, and the liquid was filtered through a 0.45-μm membrane into the sample cup prior to HPLC. An Agilent 1260 instrument equipped with an Agilent C18 4.6 × 250 mm column was used. The column temperature was set to 40°C and the flow rate was 1.0 ml/min. Mobile phase A was 20 mM sodium acetate, mobile phase B was 20 mM sodium acetate:methanol:acetonitrile at 1:2:2 (v/v), and the detection wavelength was set to 338 nm. Each sample was performed in triplicate (*n* = 3).

The nucleotides and their related compounds as well as succinic acid were extracted using a method previously reported by Bi et al. (2021). Around 3.00 g of oyster powder was homogenized in 20 ml of 8% (v/v) cold perchloric acid and ultrasonically extracted in cold water for 5 min. The supernatant was centrifuged for 20 min at 4°C and 10,000 rpm, after which the precipitate was extracted and the supernatant was pooled together. The pH was adjusted to 6.5~6.8 with 1 and 5 M potassium hydroxide, and allowed to stand for 30 min at 4°C to precipitate potassium perchlorate. The solution was centrifuged at 4°C and 10,000 rpm for 10 min, whereby the supernatant was adjusted to 25 ml with water and filtered through a 0.45-μm membrane. The filtrate was subjected to HPLC on an Agilent C18 4.6 × 250 mm column, whose temperature was set to 30°C and the flow rate to 1.0 ml/min. The mobile phase was methanol:water:phosphoric acid at 5:95:0.05 (v/v), and the detection wavelength was set to 254 nm. Each samples was performed in triplicate (*n* = 3).

For succinic acid, 3.00 g of oyster powder was homogenized in 20 ml distilled water, subjected to ultrasonic extraction for 25 min, and incubated for 30 min at 4°C. The solution was centrifuged at 4°C and 10,000 rpm for 10 min. The resulting supernatant was adjusted to 25 ml with water and filtered through a 0.45-μm membrane into the sample cup and subjected to HPLC as mentioned above for nucleotide analysis, except for detection wavelength of 210 nm.

### Volatile Organic Compounds Determination

Volatile organic compounds were determined according to the method reported by Pennarun et al. (2002). Sample processing was conducted by solid-phase microextraction. Briefly, 2.00 g of weighed sample was introduced into a 20 ml flat-bottomed headspace vial (Agilent Technologies). The extraction head (50/30 μm CAR/PDMS/DVB) was inserted into the headspace of the sample bottle, and extraction was carried out at 60°C for 30 min. After the adsorbed extraction head was removed, it was inserted into the gas chromatograph inlet and desorbed at 250°C for 3 min. Then volatile organic compounds were analyzed using a comprehensive two-dimensional gas-phase high-throughput high-resolution mass spectrometer (Pegasus GC-HRT 4D+, LECO, MI, United States) equipped with a capillary column (DB-WAX, 30 m × 0.25 × 0.25 μm) and using high-purity helium (99.999%) as the carrier gas. The oven temperature was set as follows: initial temperature 40°C for 3 min, 10°C/min to 230°C, and 230°C for 6 min. The flow rate of the carrier gas was 1 ml/min. The mass spectra conditions were as follows: electronic impact at 70 eV, emission current of 1 mA, and ion source temperature of 200°C. The C6–C26 normal alkanes were analyzed under the same chromatographic conditions as the samples, and the retention index of each substance was calculated using the instrument operating software. The content of odorant was expressed as the percentage of all volatile organic compounds.

### Data Analysis

Data are presented as mean ± SD of individual measurement. The taste activity value (TAV) was determined as the ratio of a taste substance (i.e., free amino acids, succinic acid, and nucleotides and their related compounds) in a sample relative to its taste threshold. At TAV > 1, the taste-producing substance contributed to the taste of the sample; the larger was the value, the greater was the contribution, and vice versa (Bi et al., 2021).

## Results

### Growth Performance and Proximate Composition

Growth-related traits, nutrient content, and mineral content of the two oyster species are shown in Table 1. Shell size, total weight, and soft weight were significantly larger in Portuguese oysters than in Kumamoto oysters (*p* < 0.05). Their condition index and dressing percentage were higher than those of Kumamoto oysters (*p* < 0.05). Glycogen content was much higher in Kumamoto oysters (21.8 ± 2.1%) compared to Portuguese oysters (13.7 ± 2.7%); whereas protein content (39.5 ± 2.3%) was lower than in Portuguese oysters (48.2 ± 1.6%). Moisture and lipid content were not significantly different between the two species.

**Table 1:** Production performance proximate composition and mineral content of *Crassostrea sikamea* and *Crassostrea angulata* in Xiangshan bay.

	*Crassostrea angulata*	*Crassostrea sikamea*
Shell height (mm)	59.2 ± 7.1^∗∗^	45.4 ± 5.3
Shell length (mm)	41.1 ± 6.0^∗∗^	32.4 ± 3.5
Shell width (mm)	25.4 ± 3.6^∗∗^	22.1 ± 2.9
Total weight (g)	27.9 ± 7.2^∗∗^	14.6 ± 3.2
Soft weight (g)	6.7 ± 1.8^∗∗^	3.0 ± 0.7
Dry soft weight (g)	1.03 ± 0.31^∗∗^	0.44 ± 0.14
Condition index	7.1 ± 1.4^∗∗^	5.6 ± 1.6
Dressing percentage (%)	23.2 ± 2.4^∗∗^	19.5 ± 3.4
Moisture (%)	84.6 ± 2.9	84.9 ± 3.3
Glycogen (%)	13.7 ± 2.7	21.8 ± 2.1^∗∗^
Protein (%)	48.2 ± 1.6^∗∗^	39.5 ± 2.3
Lipid (%)	8.4 ± 1.2	9.2 ± 0.9
Ca (mg/kg)	5,416 ± 747	9,367 ± 1,978^∗^
Mg (mg/kg)	2,759 ± 101	2,870 ± 193
Zn (mg/kg)	881 ± 68	1,164 ± 30^∗∗^
Cu (mg/kg)	402 ± 24	493 ± 11^∗∗^
Mn (mg/kg)	56 ± 2^∗∗^	28 ± 2
Fe (mg/kg)	640 ± 203	543 ± 158

Glycogen, protein, lipid, Ca, Mg, Zn, Cu, Mn, Fe was shown as content of dry weight. Data are the mean ± SD. *n* = 4.

∗ *p* < 0.05;

∗∗ *p* < 0.01 (Student’s *t*-test).

Ca (5416–9,437 mg/kg) was the most abundant mineral in flesh of *C. angulata* and *C. sikamea*, followed by Mg (2759–2,870 mg/kg). Generally, Kumamoto oysters contained more Ca than Portuguese oysters (*p* < 0.05); whereas Mg content did not differ significantly between the two species. Cu and Zn were also significantly higher in Kumamoto oysters (1,164 and 493 mg/kg) than in Portuguese oysters (881 and 402 mg/kg; *p* < 0.05). Fe content was not obviously different between the two species. Mn was much lower than other minerals for both species, and higher in Portuguese oysters (56 mg/kg) than Kumamoto oysters (28 mg/kg; *p* < 0.05).

### Free Amino Acids

**Figure 1:**
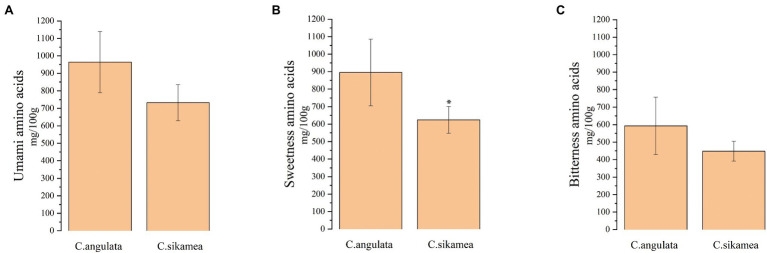
Total of flavor amino acids content of *C. sikamea* and *C. angulata* in Xiangshan bay. **(A)** A total amount of umami amino acids (sum of aspartic acid and glutamic acid); **(B)** a total amount of sweetness amino acids (sum of serine, glycine, and alanine); **(C)** a total amount of bitterness amino acids (sum of histidine, arginine, tyrosine, valine, methionine, isoleucine, leucine, and phenylalanine). Data are the mean ± SD, *n* = 4. ^∗^*p* < 0.05 (Student’s *t*-test).

As shown in Table 2, the total amount of free amino acids was higher in Portuguese oysters (5759.2 mg/100 g) than in Kumamoto oysters (4393.3 mg/100 g; *p* > 0.05). Among the 18 free amino acids measured, taurine was the most abundant (2159–2,942 mg/100 g), followed by glutamic acid (473.6–725.9 mg/100 g), alanine (325.5–317.1 mg/100 g), glycine (261.7–527.0 mg/100 g), aspartic acid (238.4–259.2 mg/100 g), and proline (201–292 mg/100 g). However, no significant difference in the content of umami amino acids (sum of aspartic acid and glutamic acid) was observed between the two species (Figure 1A). The content of sweet-flavored amino acids (sum of serine, alanine, and glycine) was significantly higher in Portuguese oysters (895 mg/100 g) than in Kumamoto oysters (624 mg/100 g; Figure 1B). The content of the bitter-flavored arginine was also significantly higher in Portuguese oysters (257.6 mg/100 g) than in Kumamoto oysters (102.1 mg/100 g), but no significant difference in the total content of bitterness amino acids (sum of histidine, arginine, tyrosine, valine, methionine, isoleucine, leucine, and phenylalanine) was detected between the two species (Figure 1C).

**Table 2:** Free amino acids, succinic acid (SA), nucleotides and their related compounds content of *C. sikamea* and *C. angulata* in Xiangshan bay.

	Content	Species		Taste threshold^#^	TAV^##^	
	(mg/100 g dry weight)	*C. angulata*	*C. sikamea*	(mg/100 ml)	*C. angulata*	*C. sikamea*
Free amino acid	Aspartic acid	238.4 ± 58.6	259.2 ± 37	100	0.37	0.39
	Glutamic acid	725.9 ± 116.3	473.6 ± 70.4^∗∗^	30	3.73	2.38
	Serine	42.8 ± 11.9	45.6 ± 6.1	150	0.04	0.05
	Alanine	325.5 ± 47.5	317.1 ± 36.4	60	0.84	0.8
	Glycine	527.0 ± 137.2	261.7 ± 36.9^∗∗^	130	0.62	0.3
	Threonine	106.6 ± 21.6	106.3 ± 16.8	260	0.06	0.06
	Arginine	257.6 ± 93.2	102.1 ± 14.3^∗^	50	0.79	0.31
	Histidine	57.8 ± 16.2	60.3 ± 5.3	20	0.45	0.46
	Tyrosine	34.6 ± 8.2	33.2 ± 3.6	—		
	Cysteine	2.3 ± 0.5	2.1 ± 0.3	—		
	Valine	44.2 ± 11.0	58.0 ± 7.2	40	0.17	0.22
	Methionine	10.3 ± 6.2	7.6 ± 1.9	30	0.05	0.04
	Phenylalanine	134.6 ± 23.3	113.6 ± 18.5	90	0.23	0.19
	Isoleucine	19.2 ± 5.1	24.0 ± 3.8	90	0.03	0.04
	Leucine	34.9 ± 9.3	49.0 ± 7.4	190	0.03	0.04
	Lysine	55.0 ± 14.8	28.8 ± 3.4^∗^	50	0.17	0.09
	Proline	201.0 ± 29.2	292.0 ± 45.1^∗^	300	0.1	0.15
	Taurine	2941.6 ± 481.4	2159.3 ± 223.4^∗^	—		
	Total FAA	5759.2 ± 1008.4	4393.3 ± 484.4			
	Succinic acid	161 ± 28	178 ± 41	37	0.67	0.73
Nucleotides and related compounds	IMP	84 ± 16	93 ± 9	25	0.52	0.56
	GMP	48 ± 9	36 ± 3	12.5	0.59	0.43
	AMP	216 ± 10	115 ± 18^∗^	50	0.67	0.35

# Taste threshold value (mg/ml) of free amino acids in water (Bi et al., 2021).

## When calculating the taste activity value (TAV), the content was converted to mg/100 g wet weight according to the moisture content.

Data are the mean ± SD, *n* = 4.

∗ *p* < 0.05;

∗∗ *p* < 0.01 (Student’s *t*-test).

### Succinic Acid and Flavor Nucleotide

Succinic acid content was higher in Kumamoto oysters (178 mg/100 g) than in Portuguese oysters (161 mg/100 g), although the difference was not significant. The nucleotide with the highest content was adenosine monophosphate (AMP), AMP was significantly more abundant in Portuguese oysters (216 mg/100 g) than in Kumamoto oysters (115 mg/100 g). No significant difference in GMP and IMP content between the two species was observed.

### Volatile Organic Compound Composition

The relative content of the compounds obtained from different samples were shown in Table 3. A total of 210–270 odorants were detected; they included aldehydes, ketones, alcohols, and alkanes. The proportion and type of odor substances differed between the two species, and some odorants were not shared in all samples (data not shown). The most abundant volatile organic compounds in both species were 3,5-octadien-2-one (33.9–42.3%), (2*E*,4*E*)-hepta-2,4-dienal (8.6–12.6%), 1-penten-3-ol (6.1–6.2%), 2-nonanone (4.3–7.2%), 3-undecen-2-one (2.9–4.8%), 2-methyl-2-pentenal (2.1–2.4%), 1,5-octadien-3-ol (1.7–2.7), and 1-octen-3-ol (1%). The relative contents of 3,5-octadien-2-one, 6-octen-2-one, and 1,5-octadien-3-ol were significantly higher in Portuguese oysters than in Kumamoto oysters (*p* < 0.05). Instead, 2-nonanone, 3-undecen-2-one, and most of the detected aldehydes were more abundant in Kumamoto oysters (*p* < 0.05). Dimethyl sulfoxide was more abundant in Portuguese oysters.

**Table 3:** Volatile organic compounds content of *Crassostrea sikamea* and *Crassostrea angulata* in Xiangshan bay.

Odors	*Crassostrea angulata*	*Crassostrea sikamea*
Ketones		
3,5-Octadien-2-one	42.3 ± 4.1	33.9 ± 4.5^∗^
2-Nonanone	4.3 ± 0.8	7.2 ± 1.1^∗∗^
3-Undecen-2-one	2.9 ± 0.6	4.8 ± 0.3^∗∗^
6-Octen-2-one	0.7 ± 0.06	0.4 ± 0.05^∗∗^
5-ethylfuran-2(5H)-one	0.7 ± 0.07	0.8 ± 0.09
3-Penten-2-one	0.6 ± 0.04	0.5 ± 0.04
3-Octen-2-one	0.4 ± 0.2	0.6 ± 0.03
3,5-Nonadien-2-one	0.4 ± 0.14	0.3 ± 0.2
2,3-Pentanedione	0.4 ± 0.1	0.3 ± 0.03
Aldehydes		
(2*E*,4*E*)-hepta-2,4-dienal	8.6 ± 1.4	12.6 ± 1.2^∗∗^
2-Methyl-2-pentenal	2.4 ± 1.1	2.1 ± 0.2
Octanal	0.7 ± 0.1	1.2 ± 0.1^∗∗^
Hexanal	0.5 ± 0.1	1.2 ± 0.3^∗∗^
Heptanal	0.3 ± 0.02	0.4 ± 0.05^∗∗^
2-Methyl-2-heptenal	0.3 ± 0.03	0.2 ± 0.04^∗^
Nonanal	0.1 ± 0.01	0.2 ± 0.03^∗^
Alcohols		
1-Penten-3-ol	6.1 ± 0.6	6.2 ± 0.9
1,5-Octadien-3-ol	2.7 ± 0.2	1.7 ± 0.5^∗^
1-Octen-3-ol	1.6 ± 0.3	1.7 ± 0.2
Others		
Dimethyl sulfoxide	0.8 ± 0.2	0.6 ± 0.2
3-Heptadecen-5-yne	5.0 ± 0.5	4.2 ± 1.5

The data are the proportion of substance in all the volatile organic compounds. Data are the mean ± SD, *n* = 4.

∗ *p* < 0.05;

∗∗ *p* < 0.01

(Student’s *t*-test).

## Discussion

### Growth Performance and Proximate Composition

Oysters are a popular type of seafood, known as “sea milk.” In this study, the growth performance and proximate composition of two oyster species, *C. sikamea* and *C. angulata*, cultivated in Xiangshan bay, China, were evaluated. Indeed, the small body size is a characteristic of Kumamoto oysters (Hedgecock et al., 1999; Melo et al., 2021). Protein, lipid, and glycogen contents of the two oyster species were comparable to those of other oyster varieties and glycogen content is believed to be related to oyster taste (Liu et al., 2020; Murata et al., 2020; Qin et al., 2020). A comparison of 31 domestic *Crassostrea gigas* strains revealed that oysters with a higher glycogen content were more often evaluated as “sweet” or “rich” (Murata et al., 2020). Although glycogen and lipid reserves are mobilized mainly to support gametogenesis (Berthelin et al., 2000), the difference in proximate nutrients between the two oyster species should not be caused by unsynchronized development of the gonads, as sampling took place in February, i.e., prior to gametogenesis. Link between higher glycogen content and better taste for oysters were found for oysters cultured in Alaska (Oliveira et al., 2006) and triploid oysters (Hong et al., 2002), thus, glycogen may contribute a lot to the special taste of Kumamoto oyster.

Oysters are good source of nutritionally important minerals although it maybe not related to oyster taste. Ca is an essential mineral for oyster and human bone health. Based on the present study, Ca content of the flesh was higher in Kumamoto oysters than in Portuguese oysters. It was also higher than in strains of Pacific oysters of different shell color (3940–5,160 mg/kg dry weight; Zhu et al., 2018) or those form two Japanese cultivation areas (2600–7,330 mg/kg dry weight; Futagawa et al., 2011). Mg content did not differ significantly between the two species measured in the present study [2759–2,870 mg/kg dry weight (425–433 mg/kg wet weight, content was converted to base on wet weight according to moisture content)]. Overall, it was higher than in rock oysters (*Crassostrea nippona*; 365 mg/kg wet weight) and Pacific oysters from Japan (334 mg/kg wet weight; Yuasa et al., 2018), but slightly lower than in Pacific oysters with differently colored shells (2920–3,710 mg/kg dry weight; Zhu et al., 2018). Cu and Zn were more abundant in Kumamoto oysters than in Portuguese oysters. Fe content (543–640 mg/kg dry weight) did not differ between oysters evaluated in the present study and commercial *C. gigas* populations (Zhu et al., 2018), but was higher than in Pacific oysters from two Japanese cultivation areas (220–373 mg/kg dry weight; Futagawa et al., 2011). The difference in mineral composition may be determined by seawater parameters and species and the higher Ca, Cu, and Zn content in Kumamoto oysters in this study may be species-specific and beneficial for its nutritional quality.

### Free Amino Acids and Nucleotides

The concentration of free amino acids is believed to be closely related to oyster taste. In this study, the content of taurine and sweetness amino acids was significantly lower in Kumamoto oysters. Taurine was the most abundant free amino acid, followed by glutamic acid, glycine, alanine, and proline, which is consistent with previous reports on 31 domestic *C. gigas* strains in Japan (Murata et al., 2020) and other oyster species or strains (Yuasa et al., 2018; Lin et al., 2019). Glutamic acid is responsible for the umami taste and has a low taste threshold. The average TAV for glutamic acid was more than 2 in both oyster species, indicating that glutamic acid greatly contributed to the umami taste of oysters. The higher proline content detected in Kumamoto oysters and previously in five oyster species collected from the Chinese coast (Lin et al., 2019) may contribute to its unique taste. Succinic acid content in this study (24.8–26.9 mg/100 g wet weight) was comparable to that observed in rock oysters and Pacific oysters (20.3–21.8 mg/100 g wet weight; Yuasa et al., 2018). Free amino acids and flavoring nucleotides are the main source of umami taste in seafood (Lin et al., 2019; Bi et al., 2021). IMP, GMP, and AMP content did not reach the taste threshold value in this study and most other oyster species (Liu et al., 2013; Yuasa et al., 2018; Bi et al., 2021). However, a synergistic effect between them may significantly increase the umami taste. These results indicate that free amino acid and flavor nucleotides may not the source of special taste of Kumamoto oyster.

### Volatile Organic Compounds

In this study, most volatile organic compounds were shared in both Kumamoto and Portuguese oysters. The most abundant compounds were ketones, followed by aldehydes and alcohols (Table 3), which reflected the characteristic odors of fresh oysters, such as cucumbers and mushrooms. In contrast, aldehydes appear to be dominant in oysters along the Chinese coast (Lin et al., 2019), and alcohols in European flat oysters and Pacific oysters (Pennarun et al., 2002; van Houcke et al., 2016). The type and proportion of volatile organic compounds vary greatly between different regions and species (Kawabe et al., 2019; Lin et al., 2019; Soares et al., 2020). Some of the compounds such as 3,5-octadien-2-one, which gives a fatty fruity odor, were detected previously (Pennarun et al., 2002), but were more abundant in the present study, and that is maybe the basal odor of two oyster species in the local bay. Other compounds included (2*E*,4*E*)-hepta-2,4-dienal, octanal, hexanal, and heptanal, which give a mushroom, moss, and green odor (van Houcke et al., 2016; Kawabe et al., 2019), 1-octen-3-ol, 1-penten-3-ol, and 1,5-octadien-3-ol, which give a fresh mushroom odor (van Houcke et al., 2016), and dimethyl sulfoxide, which gives an unpleasant smell (Lin et al., 2019) were also shared. The threshold for aldehydes was extremely low, while that for ketones and alcohols was somewhat higher. Previously results shown that 11 of the 16 key volatile components found in oysters sampled from 12 sites along the Chinese coast were aldehydes (Lin et al., 2019). Thus, the higher proportion of aldehydes may contribute to the special odor of Kumamoto oyster.

As mentioned, the nutrient composition varied among culture conditions (Pennarun et al., 2003; van Houcke et al., 2017; Bi et al., 2021), in this study, two oyster species from only one cultured sea area and one sampling time were analyzed and the results has limited representation. In follow-up study, Kumamoto oyster sampling at multiple sea areas and different months or seasons are necessary to compare whether the nutrients content are still higher of Kumamoto oysters detected in this study for better understanding the molecular basis of the its special taste.

To summarize, based on the present study, Kumamoto oysters are rich in glycogen, Ca, Zn, Cu, and aldehydes, of which glycogen content may contribute to their characteristic sweet taste, Ca, Zn, Cu contribute to its mineral nutritional value and aldehydes may represent the characteristic odors. However, free amino acid and flavor nucleotides may not the source of special taste of Kumamoto oyster. These results provide more data on the molecular basis underlying the unique taste of Kumamoto oysters and will help maximize the utilization of local oyster germplasm resources.

## Data Availability Statement

The original contributions presented in the study are included in the article/Supplementary Material, further inquiries can be directed to the corresponding author.

## Author Contributions

QX and ZL conceived and designed the study. SL, HX, and SJ collected and sampled the oysters. SJ conducted the oyster species identification. SL and HX performed the statistical analyses. SL and QX wrote and revised the manuscript. All authors contributed to the article and approved the submitted version.

## Conflict of Interest

The authors declare that the research was conducted in the absence of any commercial or financial relationships that could be construed as a potential conflict of interest.

## Publisher’s Note

All claims expressed in this article are solely those of the authors and do not necessarily represent those of their affiliated organizations, or those of the publisher, the editors and the reviewers. Any product that may be evaluated in this article, or claim that may be made by its manufacturer, is not guaranteed or endorsed by the publisher.

## Supplementary Material

Supplementary Figure 1 The flesh color comparison of *Crassostrea sikamea* and *Crassostrea angulata* in Xiangshan bay and species identification by molecular method. **(A)** The yellow-green flesh of Kumamoto oyster (left) and white flesh of Portuguese oyster (right). **(B)** Lane 1–4, specific bands for Portuguese oyster around 750 bp, lane 6–9, specific bands for Portuguese oyster around 660 bp, and lane 5: DL2000 DNA marker with the brightest band (750 bp).
