# Supplementary figures and images for: Molecular Basis of Taste and Micronutrient Content in Kumamoto Oysters (Crassostrea Sikamea) and Portuguese Oysters (Crassostrea Angulata) From Xiangshan Bay
Source: Front Physiol. 2021 Jul 27;12:713736. doi: 10.3389/fphys.2021.713736 (PMC8353274; doi:10.3389/fphys.2021.713736)

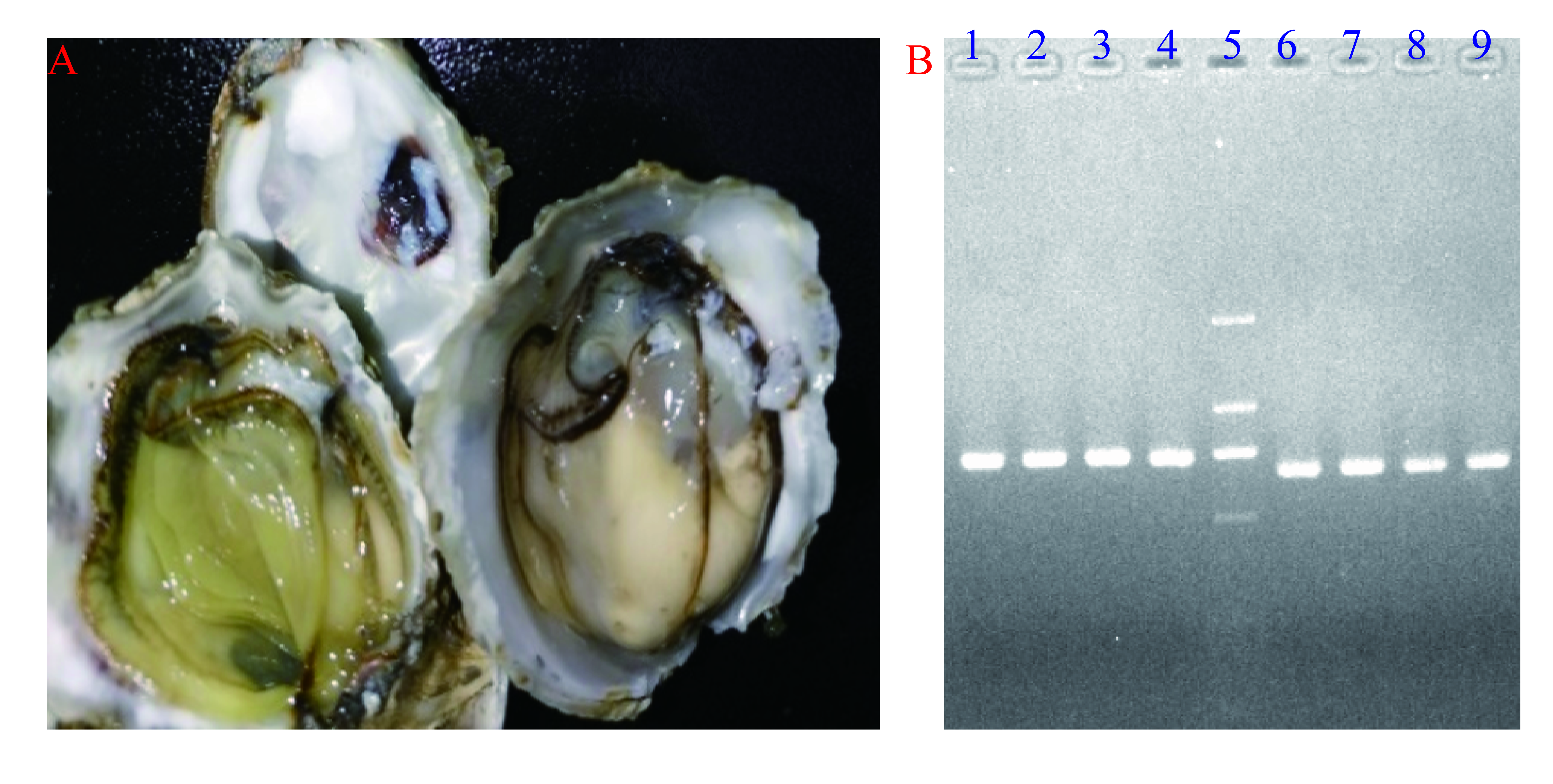

Supplement: Supplementary Figure 1 — The flesh color comparison of Crassostrea sikamea and Crassostrea angulata in Xiangshan bay and species identification by molecular method. (A) The yellow-green flesh of Kumamoto oyster (left) and white flesh of Portuguese oyster (right). (B) Lane 1–4, specific bands for Portuguese oyster around 750 bp, lane 6–9, specific bands for Portuguese oyster around 660 bp, and lane 5: DL2000 DNA marker with the brightest band (750 bp). [file Image_1.TIF]
